# Identification of priority pathogens for aetiological diagnosis in adults with community-acquired pneumonia in China: a multicentre prospective study

**DOI:** 10.1186/s12879-023-08166-3

**Published:** 2023-04-14

**Authors:** Lulu Zhang, Yan Xiao, Guoliang Zhang, Hongru Li, Jianping Zhao, Mingwei Chen, Fuhui Chen, Ling Liu, Yalun Li, Liping Peng, Feng Zhao, Donghong Yang, Zhongmei Wen, Lei Wu, Shuo Wu, Yajiao Sun, Ying Wang, Lan Chen, Xinming Wang, Lihui Wang, Weimin Li, Haibo Qiu, Yusheng Chen, Zhancheng Gao, Lili Ren, Jianwei Wang

**Affiliations:** 1 Institute of Pathogen Biology, Chinese Academy of Medical Sciences & Peking Union Medical College, No.9 Dong Dan San Tiao, Dongcheng District, Beijing, 100730 P.R. China; 2 Key Laboratory of Respiratory Disease Pathogenomics, Chinese Academy of Medical Sciences, Peking Union Medical College, No.9 Dong Dan San Tiao, Dongcheng District, Beijing, 100730 P.R. China; 3 Shenzhen Third People’s Hospital, Shenzhen, 518112 P.R. China; 4 Fujian Provincial Hospital, Fujian, 350001 P.R. China; 5 Tongji Hospital, Tongji Medical College of Hust, Wuhan, 430030 P.R. China; 6 The First Affiliated Hospital of Xi’an Jiaotong University, Xi’an, 710061 P.R. China; 7 The Second Affiliated Hospital of Harbin Medical University, Harbin, 150001 P.R. China; 8 Jiangsu Provincial Key Laboratory of Critical Care Medicine, Department of Critical Care Medicine, School of Medicine, Zhongda Hospital, Southeast University, Nanjing, 210009 P.R. China; 9 Department of Respiratory and Critical Care Medicine, Lung Cancer Treatment Center, West China Hospital, Sichuan University, Chengdu, 610041 P.R. China; 10 Department of Respiratory Medicine, The First Hospital of Jilin University, Changchun, 130021 China; 11 Xijing Hospital, Fourth Military Medical University, Xi’an, 710032 P.R. China; 12 Peking University People’s Hospital, No.11 Xizhimen South Dajie, Xicheng District, Beijing, 100044 P.R. China

**Keywords:** Community-acquired pneumonia, Aetiology, Risk factor, Severe infection, Priority screening pathogens

## Abstract

**Background:**

Community-acquired pneumonia (CAP) is a major public health challenge worldwide. However, the aetiological and disease severity-related pathogens associated with CAP in adults in China are not well established based on the detection of both viral and bacterial agents.

**Methods:**

A multicentre, prospective study was conducted involving 10 hospitals located in nine geographical regions in China from 2014 to 2019. Sputum or bronchoalveolar lavage fluid (BALF) samples were collected from each recruited CAP patient. Multiplex real-time PCR and bacteria culture methods were used to detect respiratory pathogens. The association between detected pathogens and CAP severity was evaluated.

**Results:**

Among the 3,403 recruited eligible patients, 462 (13.58%) had severe CAP, and the in-hospital mortality rate was 1.94% (66/3,403). At least one pathogen was detected in 2,054 (60.36%) patients, with two or more pathogens were co-detected in 725 patients. The ten major pathogens detected were *Mycoplasma pneumoniae* (11.05%), *Haemophilus influenzae* (10.67%), *Klebsiella pneumoniae* (10.43%), influenza A virus (9.49%), human rhinovirus (9.02%), *Streptococcus pneumoniae* (7.43%), *Staphylococcus aureus* (4.50%), adenovirus (2.94%), respiratory syncytial viruses (2.35%), and *Legionella pneumophila* (1.03%), which accounted for 76.06–92.52% of all positive detection results across sampling sites. *Klebsiella pneumoniae* (*p* < 0.001) and influenza viruses (*p* = 0.005) were more frequently detected in older patients, whereas *Mycoplasma pneumoniae* was more frequently detected in younger patients (*p* < 0.001). Infections with *Klebsiella pneumoniae*, *Staphylococcus aureus*, influenza viruses and respiratory syncytial viruses were risk factors for severe CAP.

**Conclusions:**

The major respiratory pathogens causing CAP in adults in China were different from those in USA and European countries, which were consistent across different geographical regions over study years. Given the detection rate of pathogens and their association with severe CAP, we propose to include the ten major pathogens as priorities for clinical pathogen screening in China.

**Supplementary Information:**

The online version contains supplementary material available at 10.1186/s12879-023-08166-3.

## Background

Community-acquired pneumonia (CAP) is a major public health challenge worldwide, with 2.6 million deaths in 2019 [1–4]. Multiple respiratory pathogens are known to cause CAP. Influenza virus (IFV), human parainfluenza viruses (HPIVs), respiratory syncytial virus (RSV), human enterovirus (EV)/rhinovirus (HRV), human adenonvirus (AdV), human coronavirus (HCoV) and human metapeumovirus (HMPV) are the major viruses in CAP; while *Streptococcus pneumoniae* (*S. pneumoniae*), *Haemophilus influenzae* (*H. influenzae*), *Mycoplasma pneumoniae* (*M. pneumoniae*), and *Legionella pneumophila* (*L. pneumophila*) are common respiratory bacteria in CAP [5–8]. Moreover, the aetiology of CAP varies geographically due to the impact of social, economic, environmental and demographic factors [7–9].

Due to the complexity of CAP aetiology, the implementation of multiplex molecular detection to figure out the incidence of major pathogens has significantly improved our understanding of CAP aetiology [3, 5, 6]. However, routine screening of respiratory viruses other than IFVs is only recommended in patients with severe CAP and immunodeficiency [10]. More sufficient laboratory-based aetiological evidence is essential to improve the understanding of the pathogenesis of CAP and to develop effective guidelines for aetiological diagnosis and anti-infective therapies to CAP.

The aetiology of CAP in adults and its association with adverse outcomes in patients in China, one of the most populous countries in the world that is undergoing rapid industrialization, urbanization and ageing, have not been well defined based on a large-scale comprehensive aetiological study of both viral and bacterial pathogens by multiplex detection. Here, we conducted a multicentre prospective study to determine the aetiology of CAP in adults in China. We also evaluated the association between respiratory pathogens and disease severity and identified priority pathogens for screening.

## Methods

### Study design and participants

From 1 to 2014 to 31 December 2019, hospitalized CAP patients aged ≥ 14 years were recruited from 10 hospitals in nine cities located in different geographical regions in mainland China. CAP was diagnosed according to the 2007 guidelines of the American Thoracic Society [11]. Detailed inclusion and exclusion criteria are provided in the supplementary materials (Additional file 1: Supplementary Methods). Demographic and clinical information were obtained from clinical records.

### Specimen collection and respiratory pathogen detection

For each patient, sputum or bronchoalveolar lavage fluid (BALF) and whole blood samples were collected at enrolment. For a few patients, pleural effusion and endotracheal aspirate samples were collected. A total of 200 µl samples were used for nucleic acid extraction as previously reported by using easyMAG (bioMerieux, Marcy l’Etoile, France) [9, 12, 13]. Thirty-three respiratory pathogens were detected by using the multiplex real-time PCR method (Fast Track Diagnostic, Junglinster, Luxembourg), including IFVs (A, B and C) and the 2009 subtype of IFVA/H1N1, HPIV 1–4, HCoVs (NL63, 229E, OC43 and HKU1), HMPV A and B, EVs (including HRVs), RSV A and B, Adv, human parechovirus (HPeV), human bocavirus (HBoV), *Pneumocystis jirovecii* (*P. jirovecii*), *M. pneumoniae*, *Chlamydophila pneumoniae* (*C. pneumoniae*), *S. pneumoniae*, *H. influenzae*, *H. influenzae* type b (Hib), *Staphylococcus aureus* (*S. aureus*), *Moraxella catarrhalis* (*M. catarrhalis*), *Bordetella* spp. (except for *Bordetella parapertussis*), *Klebsiella pneumoniae* (*K. pneumoniae*), *L. pneumophila* and *Salmonella* spp. In addition, bacterial culture testing was performed on some specimens. Positive results were considered in further analysis.

### Statistical analysis

The Wilcoxon rank sum test or *t* test was used to analyse continuous variables. The chi-square test or Fisher’s exact test was used to analyse categorical variables. Multivariate logistic regression was used to evaluate the association between aetiological factors and severe CAP adjusted by age, sex, season, duration of illness, previous antibiotic exposure (defined as antibiotic use within 5 days prior to admission) and underlying diseases. A two-sided *p* < 0.05 was considered statistically significant. All statistical analyses were conducted by using IBM SPSS (v.19.0; IBM Corp., Armonk, NY, USA) and the R package (version 3.6.1) [14].

The association of the detected pathogens with the risk of severe pneumonia was calculated using the adjusted odds ratio (OR). The aetiological estimation fraction was determined based on the detection rate of pathogens and their risk associations with severe pneumonia. The ten major pathogens were identified by the aetiological estimated fraction. The calculation form of the aetiological estimation fraction is as follows:

$$\begin{array}{c}{\rm{Aetiological}}\,{\rm{estimated}}\,{\rm{fraction}}\,(\% )\,{\rm{ = }}\,\\{\rm{Detection}}\_{\rm{rate}}\,(\%)\,\times\,{\rm{Severe}}\_{\rm{OR}}\end{array}$$

## Results

### Patient characteristics

A total of 3,403 hospitalized adults with CAP were enrolled in this study. Of which 462 (13.58%) had severe pneumonia, 317 (9.32%) required intensive care, and 66 (1.94%) died in the hospital (Table 1). The median age of the patients was 58 (interquartile range, 40–70) years. There were 954 (6.61%) patients with underlying diseases, and the most common clinical symptoms were cough (3,005, 88.30%), sputum (2,552, 74.99%), dyspnoea (442, 12.99%) and chest pain (437, 12.84%) (Table 1). The number of cases from each site ranged from 104 to 599 (Additional file 2: Table S1).

**Table 1:** Demographic and clinical characteristics of enrolled patients with community-acquired pneumonia requiring hospitalization

	All cases	Virus positive	Bacterium positive	Negative detection	P-value (virus vs. bacterium)	P-value (virus vs. negative)
**Total**	3403	654^a^	948^b^	1349		
**Age group, no. (%)**	-					
14–24 yrs	231 (6.79)	30 (4.59)	103 (10.86)	71 (5.26)	**< 0.001**	0.517
25–44 yrs	795 (23.36)	129 (19.72)	282 (29.75)	265 (19.64)	**< 0.001**	0.966
45–64 yrs	1161 (34.12)	228 (34.86)	261 (27.53)	523 (38.77)	**0.002**	0.090
≥ 65 yrs	1216 (35.73)	267 (40.83)	302 (31.86)	490 (36.32)	**< 0.001**	0.051
**Sex (male/female)**	2060/1343	397/257	547/401		0.23	0.421
**Season, no. (%)**	-					
Spring	799 (23.48)	165 (25.23)	214 (22.57)	326 (24.17)	0.219	0.604
Summer	753 (22.13)	102 (15.60)	253 (26.69)	308 (22.83)	**< 0.001**	**< 0.001**
Autumn	911 (26.77)	151 (23.09)	290 (30.59)	350 (25.95)	**0.001**	0.166
Winter	940 (27.62)	236 (36.09)	191 (20.15)	365 (27.06)	**< 0.001**	**< 0.001**
**Median duration of illness (days)**	7 (4–11)	7 (4–10)	7 (3–10)		0.167	
**Died in hospital, no. (%)**	66 (1.94)	16 (2.45)	22 (2.32)	17 (1.26)	0.871	0.050
**Severe pneumonia, no. (%)**	462 (13.58)	121 (18.50)	123 (12.97)	151 (11.19)	**0.002**	**< 0.001**
**ICU admission, no. (%)**	317 (9.32)	84 (12.84)	88 (9.28)	104 (7.71)	**0.024**	**< 0.001**
**Sepsis, no. (%)**	116 (3.41)	26 (3.98)	37 (3.90)	27 (2.00)	0.941	**0.010**
**Noninvasive ventilation**	374 (10.99)	94 (14.37)	90 (9.49)	138 (10.23)	**0.003**	**0.007**
**Invasive ventilation**	165 (4.85)	37 (5.66)	57 (6.01)	48 (3.56)	0.766	**0.029**
**Underlying diseases, no. (%)**	-					
Diabetes	238 (6.99)	46 (7.03)	64 (6.75)	94 (6.97)	0.826	0.957
Congestive heart failure	226 (6.64)	61 (9.33)	48 (5.06)	84 (6.23)	**0.001**	**0.012**
Cerebral vascular disease	164 (4.82)	34 (5.20)	45 (4.75)	67 (4.97)	0.681	0.824
COPD	99 (2.91)	24 (3.67)	19 (2.00)	41 (3.04)	**0.043**	0.455
Chronic liver disease	79 (2.32)	19 (2.91)	23 (2.43)	29 (2.15)	0.555	0.300
Chronic kidney disease	56 (1.65)	13 (1.99)	16 (1.69)	17 (1.26)	0.658	0.209
Bronchiectasis	37 (1.09)	9 (1.38)	13 (1.37)	12 (0.89)	0.993	0.316
**Clinical symptoms, no. (%)**	-					
Cough	3005 (88.30)	593 (90.67)	842 (88.82)	1153 (85.47)	0.233	**0.001**
Sputum	2552 (74.99)	502 (76.76)	715 (75.42)	991 (73.46)	0.538	0.112
Dyspnoea	442 (12.99)	99 (15.14)	98 (10.34)	174 (12.90)	**0.004**	0.171
Chest pain	437 (12.84)	78 (11.93)	109 (11.50)	189 (14.01)	0.793	0.198
Tachypnea	398 (11.70)	90 (13.76)	94 (9.92)	151 (11.19)	**0.018**	0.098
Short breath	396 (11.64)	99 (15.14)	74 (7.81)	185 (13.71)	**< 0.001**	0.392
Fatigue	238 (6.99)	43 (6.57)	62 (6.54)	97 (7.19)	0.978	0.612
Chills	232 (6.82)	60 (9.17)	53 (5.59)	90 (6.67)	**0.006**	**0.046**
Sore throat	220 (6.46)	37 (5.66)	85 (8.97)	55 (4.08)	**0.014**	0.113
Headache	176 (5.17)	31 (4.74)	66 (6.96)	66 (4.89)	0.067	0.882
Runny nose	149 (4.38)	34 (5.20)	48 (5.06)	37 (2.74)	0.904	**0.005**
Haemoptysis	144 (4.23)	33 (5.05)	39 (4.11)	60 (4.45)	0.376	0.551
Myalgia	130 (3.82)	29 (4.43)	32 (3.38)	50 (3.71)	0.276	0.433
Nausea	90 (2.64)	21 (3.21)	27 (2.85)	32 (2.37)	0.675	0.273
Diarrhoea	33 (0.97)	10 (1.53)	8 (0.84)	9 (0.67)	0.201	0.062
Arthralgia	30 (0.88)	7 (1.07)	6 (0.63)	14 (1.04)	0.337	0.947
Abdominal pain	27 (0.79)	6 (0.92)	9 (0.95)	8 (0.59)	0.948	0.405
Oliguria	11 (0.32)	4 (0.61)	2 (0.21)	4 (0.30)	0.233	0.287
Bleeding	9 (0.26)	3 (0.46)	2 (0.21)	3 (0.22)	0.404	0.399
**Laboratory findings on admission, no. (%)**	-					
Temperature ≥ 38·0 °C	1502 (44.14)	310 (47.40)	422 (44.51)	554 (41.07)	0.254	**0.007**
Elevated WBC count (> 10 × 10^9/L)	846 (28.01)^c^	163 (27.30)^d^	243 (30.04)^e^	197 (14.60)	0.264	0.079
ALT > 40, U/L	669 (22.17)^f^	135 (22.73)^g^	163 (20.10)^h^	318 (23.57)	0.234	0.596
BUN > 7, mmol/L	623 (20.88)^i^	137 (23.26)^j^	163 (20.25)^k^	292 (21.65)	0.177	**0.005**
PaO_2_/FiO_2_ < 300, mmHg	2745 (98.95)^l^	492 (90.61)^m^	671 (87.26)^n^	240 (17.79)	0.059	**< 0.001**

^a^ Virus-positive patients are those with one or more positive detection results for viruses, not including those with co-detection of viruses and bacteria

^b^ Bacterium-positive patients are those with one or more positive detection results for bacteria, not including those with co-detection of bacteria and viruses

^c^ Information on WBC count was missing for 383 patients; therefore, the total number for this item was 3,020

^d^ The total number for this item was 597

^e^ The total number for this item was 809

^f^ Information on ALT was missing for 386 patients; therefore, the total number for this item was 3,017

^g^ The total number for this item was 594

^h^ The total number for this item was 811

^i^ Information on BUN was missing for 419 patients; therefore, the total number for this item was 2,984

^j^ The total number for this item was 589

^k^ The total number for this item was 805

^l^ Information on PaO_2_/FiO_2_ was missing for 629 patients; therefore, the total number for this item was 2,774

^m^ The total number for this item was 543

^n^ The total number for this item was 769

ICU, intensive care unit; COPD, chronic obstructive pulmonary disease; WBC, white blood cell, elevated white blood cell (WBC) count was defined as greater than 10 × 10^9^ cells per L for adults; ALT, alanine aminotransferase; BUN, blood urea nitrogen

Among the 3,123 patients with available antibiotic exposure data before admission, 720 (22.99%) patients had antibiotic exposure before admission. The most common antibiotics were β-lactams (351, 11.21%), followed by quinolones (232, 7.41%), macrolides (39, 1.25%) and other drugs (207, 6.61%). A higher proportion of antibiotic exposure before admission was found in patients with severe CAP than in those with non-severe CAP (37.12% vs. 20.94%, *p* < 0.001) (Additional file 3: Table S2).

### Detection of respiratory pathogens

A total of 3,213 eligible sputum, 190 BALF, 656 blood, 13 pleural effusion and 12 endotracheal aspirate samples were collected in the study. Molecular test results were available for all 3,403 respiratory specimens. In parallel, bacterial culture testing was performed on 1,001 (54, 5.39%) sputum, 63 (2, 3.17%) BALF, 260 (9, 3.46%) blood, 11 (0, 0%) pleural effusion and 9 (2, 22.22%) endotracheal aspirate samples. All positive results were included in further aetiological evaluation. At least one pathogen was detected in 2,054 (60.36%) patients; specifically, a single pathogen was detected in 1,329 (39.05%) patients, and multiple pathogens were detected in 725 (21.30%) patients.

**Fig. 1:**
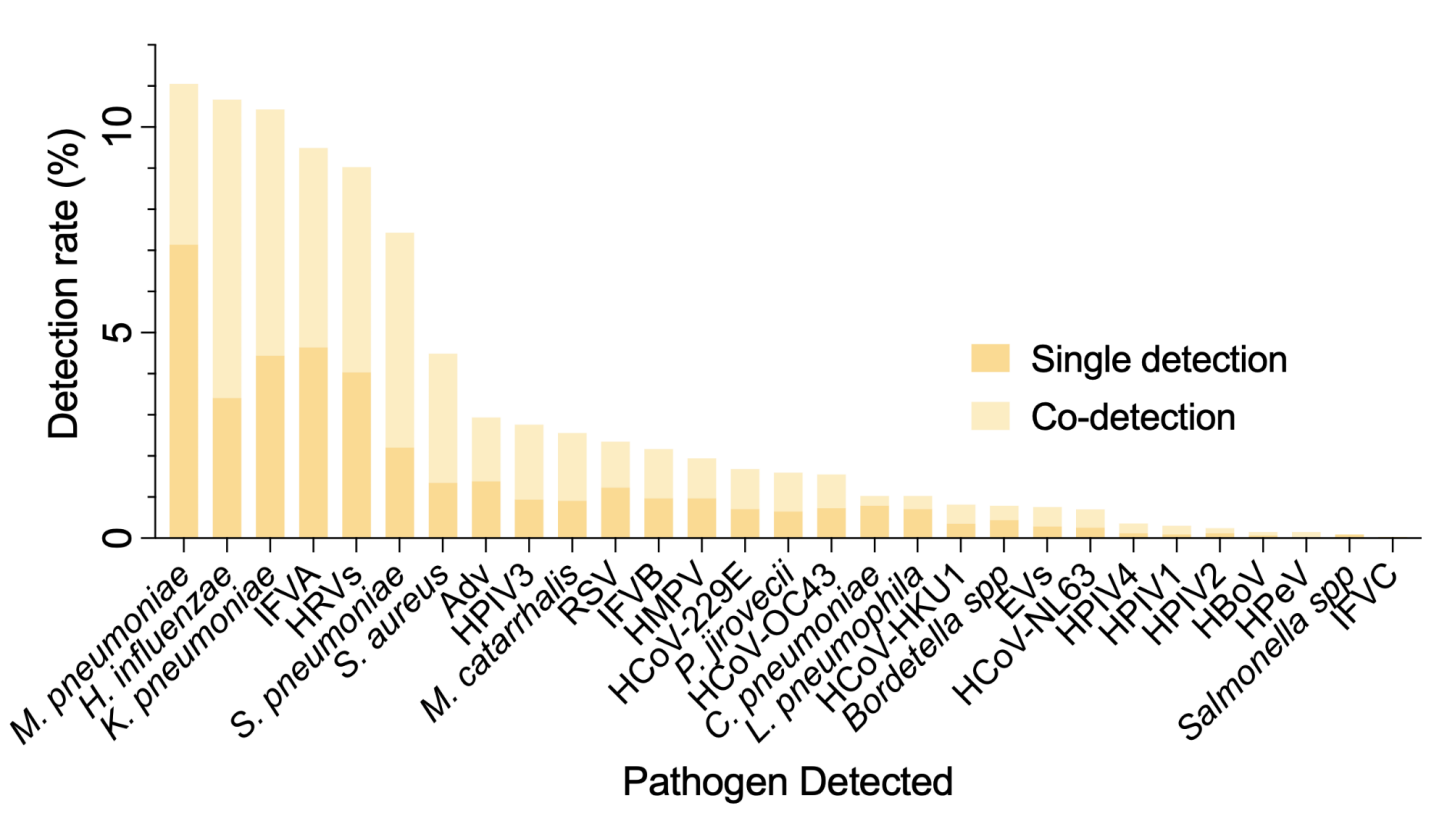
**Pathogen detection among Chinese adults with community-acquired pneumonia requiring hospitalization, 2014–2019.** The dark shade represents single detection, while the light shade represents co-detection. IFVs, influenza viruses (A, B and C); EVs, enteroviruses; HRVs, human rhinoviruses; HCoVs, human coronaviruses; HPIVs, parainfluenza viruses; Adv, adenovirus; RSV, respiratory syncytial virus; HMPV, human metapneumovirus; HBoV, human bocavirus; HPeV, human parechovirus; *M. pneumoniae, Mycoplasma pneumoniae; K. pneumoniae, Klebsiella pneumoniae; H. influenzae, Haemophilus influenzae; S. pneumoniae, Streptococcus pneumoniae; S. aureus, Staphylococcus aureus; M. catarrhalis, Moraxella catarrhalis; P. jirovecii, Pneumocystis jirovecii; C. pneumonia, Chlamydia pneumoniae; L. pneumophila, Legionella pneumophila/Legionella longbeachae; Bordetella* spp., *Bordetella* species (except for *Bordetella parapertussis*); *Salmonella* spp., *Salmonella* species

*M. pneumoniae* (376, 11.05%), *H. influenzae* (363, 10.67%), *K. pneumoniae* (355, 10.43%), IFVA (323, 9.49%), HRVs (307, 9.02%), *S. pneumoniae* (253, 7.43%), *S. aureus* (153, 4.50%) and Adv (100, 2.94%) were the most frequently detected pathogens, accounting for more than 80% of all positive results (Fig. 1, Additional file 4: Table S3). The detection rate of remnant respiratory pathogens was less than 3.00%. Regardless of the viral subtype, IFVs (A, B and C) (398, 11.70%) were the most frequently detected pathogens (Additional file 5: Figure S1). The overall pathogen detection rate varied from 37.23 to 79.09% among the nine sites during the study period (Additional file 6: Table S4).

Compared with bacterium-positive patients (n = 948) and pathogen-negative patients (n = 1,349), virus-positive patients (n = 654) had a higher rate of chills (*p* = 0.006, *p* = 0.046), severe CAP (*p* = 0.002, *p* < 0.001), ICU admission (*p* = 0.024, *p* < 0.001), and noninvasive ventilation (*p* = 0.003, *p* = 0.007). When underlying disease was taken into account, virus-positive patients had a higher rate of congestive heart failure than patients positive for bacteria and those with negative detection results (*p* = 0.001, *p* = 0.012, respectively) (Table 1).

### Age distribution of patients with respiratory infections

**Fig. 2:**
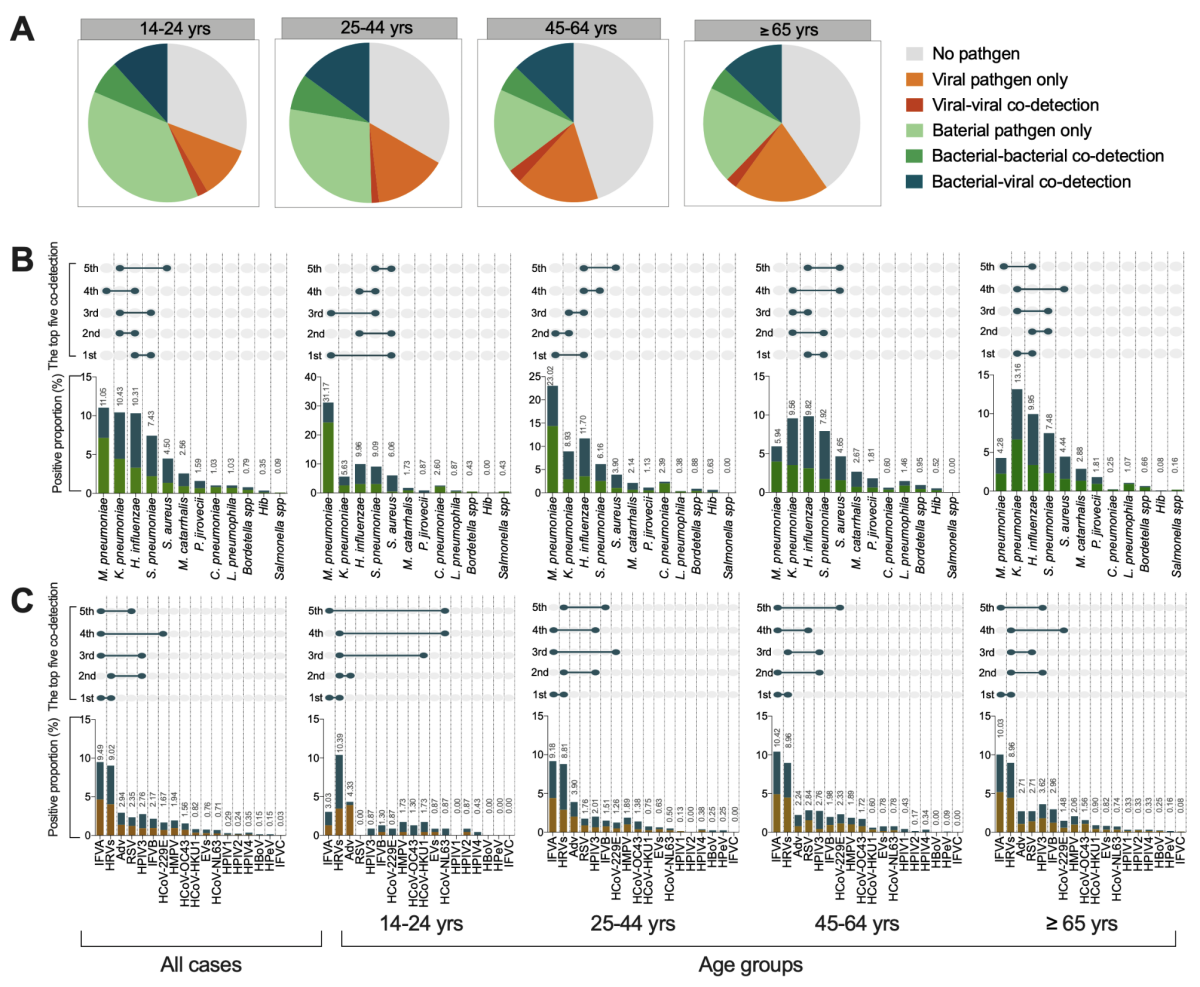
**Age distribution of community-acquired pneumonia patients infected with respiratory pathogens.** (A) The detection rate of viruses and bacteria in different age groups. (B) Positive detection of bacteria in different age groups. (C) Positive detection of viruses in different age groups. The green bar indicates single bacterium detection, the orange bar indicates single virus detection, and the dark green bar indicates co-detection of bacteria and viruses

To identify the age distribution of patients with infections, we categorized four age groups as 14–24, 25–44, 45–64 and ≥ 65 years to compare the frequency of positive detection. The highest positive rates of detection of total pathogens (69.26%) and bacteria (56.28%) were found in the 14–24 age group (chi-square test, *p* < 0.001). While the virus-positive rate was highest in the elderly aged ≥ 65 years old (34.87%, chi-square test, *p* = 0.017) (Fig. 2A). For each pathogen, higher frequencies of *M. pneumoniae* and *C. pneumoniae* were found in the 14–24 year-old group (*p* < 0.001), IFVA (*p* = 0.005) and RSV (*p* = 0.034) in the 45–64 year-old group, and HPIV3 (*p* = 0.044) and *K. pneumoniae* (*p* < 0.001) in the ≥ 65 year-old group (chi-square test) (Fig. 2B and C, Additional file 7: Table S5). The detection rate of the remaining screened pathogens showed no significant difference among age groups.

### Temporal distribution of respiratory pathogens

**Fig. 3:**
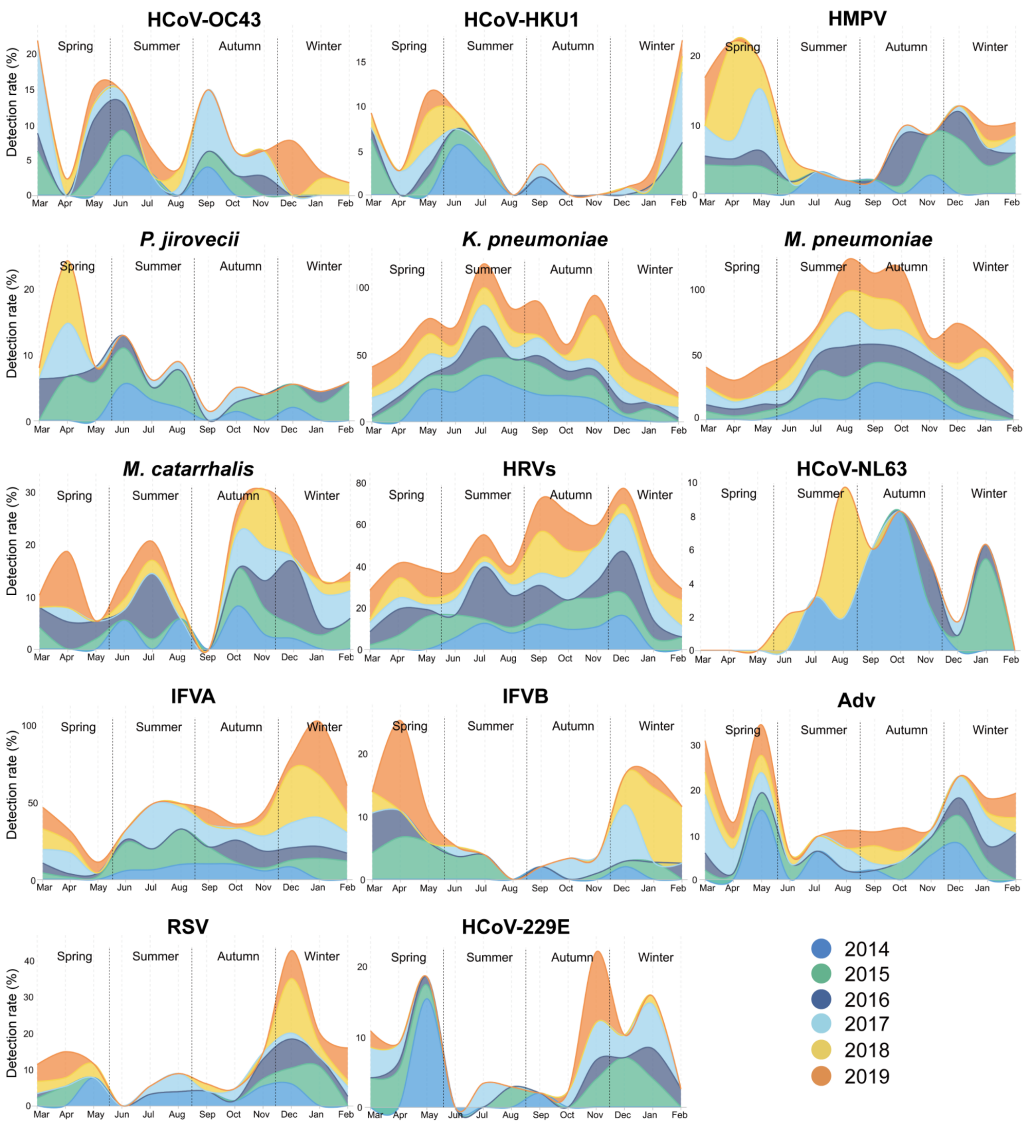
**Temporal distribution of community-acquired pneumonia in patients with respiratory pathogen infections.** The area under the curves represents monthly detection rates. The horizontal axis shows the variation in pathogen detection between seasons. The seasons were defined as spring (March to May), summer (June to August), autumn (September to November), and winter (December to February). Data between years (2014–2019) are shown in different colours

The pathogen detection rates varied during the study period, with the highest rate in 2014 (72.48%) and the lowest in 2016 (45.53%) (Additional file 8: Table S6). Although the detection rate of each pathogen fluctuated slightly, the most commonly detected pathogens were relatively consistent, with eight pathogens ranking in the top 10 every year. Seasonality was analysed based on the peak detection rate of each pathogen, and 10 viruses and 4 bacteria were found to have significant seasonality, for example, *K. pneumoniae* peaks in summer (June to August), while IFVA and RSV in winter (December to February) (Fig. 3, Additional file 9: Table S7).

### Co-detection of respiratory pathogens

Two or more (termed “multiple” hereafter) pathogens were co-detected in 725 (21.30%) patients, namely, multiple viruses in 78 (2.29%) patients, multiple bacteria in 195 (5.73%), and viruses with bacteria in 452 (13.28%) patients (Additional file 4: Table S3). Two pathogens were found in 542 (74.76%) patients, three in 145 (19.28%), and four or more in 38 (5.24%). The most common co-detected pathogens were IFVA with *H. influenzae* (n = 53), *S. pneumoniae* with *H. influenzae* (n = 51) and *K. pneumoniae* with *H. influenzae* (n = 42) (Additional file 10: Figure S2).

### Associations of pathogens with severe CAP

**Fig. 4:**
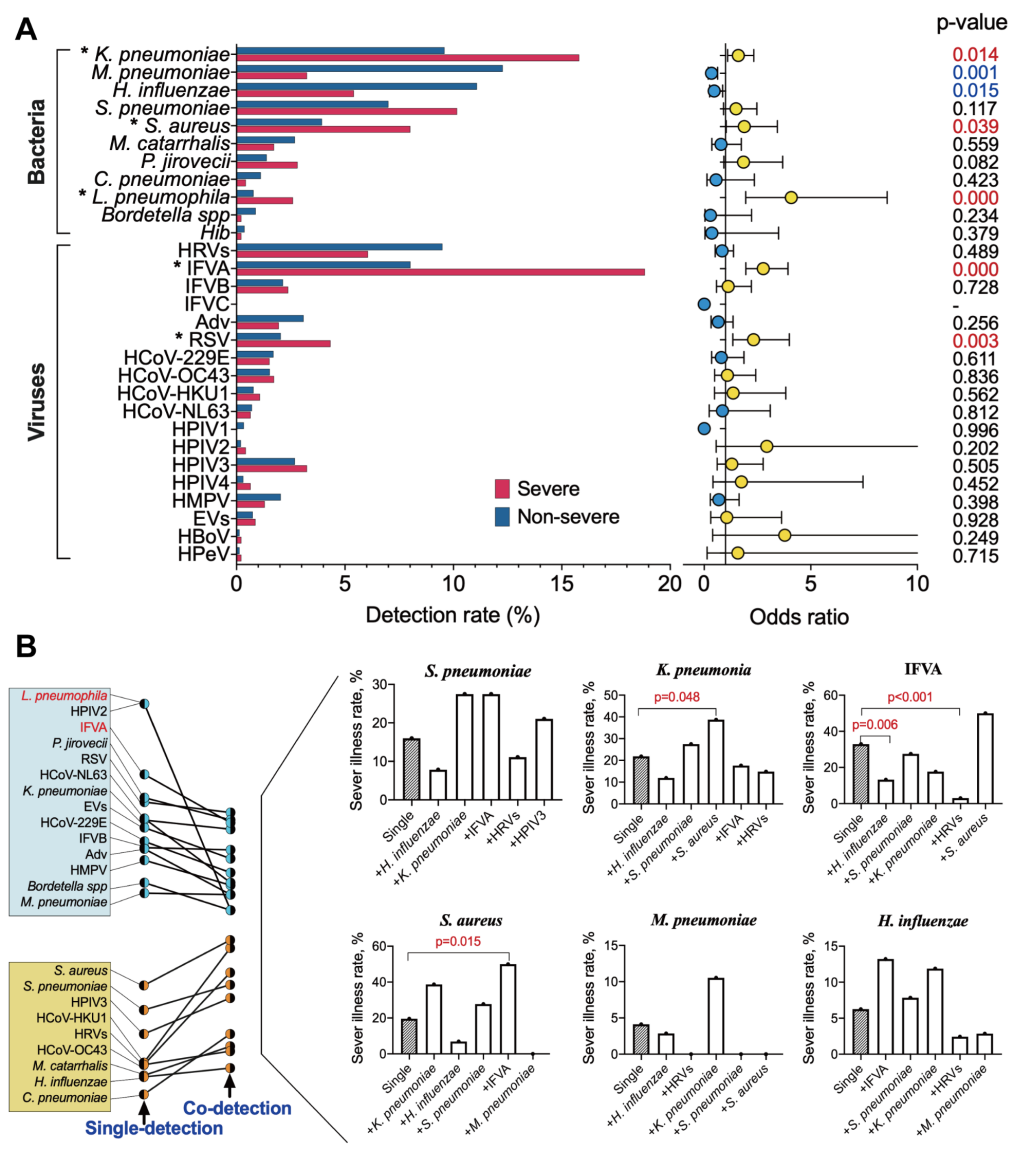
**Associations of respiratory pathogens with severe community-acquired pneumonia (CAP).** (A) The detection rate of respiratory pathogens and adjusted odds ratios (ORs) in patients with severe CAP and non-severe CAP. ORs adjusted for age (years), sex, season, duration of illness (days), previous antibiotic exposure and underlying diseases. (B) Comparison of the effects of multiple detections and single detection on severe disease

The overall positive detection rate was significantly higher in patients with severe (67.32%) versus non-severe CAP (59.27%) (chi-square test, *p* = 0.001). *K. pneumoniae* (OR 1.599, 95% confidence interval [CI] 1.099–2.327, *p* = 0.014), *S. aureus* (OR 1.883, 95% CI 1.032–3.434, *p* = 0.039), *L. pneumophila* (OR 4.086, 95% CI 1.946–8.582, *p* < 0.001), IFVA (OR 2.771, 95% CI 1.954–3.930, *p* < 0.001) and RSV (OR 2.315, 95% CI 1.343–3.992, *p* = 0.003) were related to severe CAP after adjusting for the confounding factors of age, sex, season, days post illness onset, prior antibiotic exposure and underlying diseases (Fig. 4A). We also found that the co-detection of IFVA with *S. aureus* was more frequent in patients with severe CAP than single *S. aureus* detection (chi-square test, *p* = 0.015) and that co-detection of *K. pneumoniae* with *S. aureus* was more frequent in patients with severe CAP than single *K. pneumoniae* detection (chi-square test, *p* = 0.048) (Fig. 4B). Such findings further indicate the important role of pathogens, for example, IFVA and *S. aureus*, in severe CAP.

**Fig. 5:**
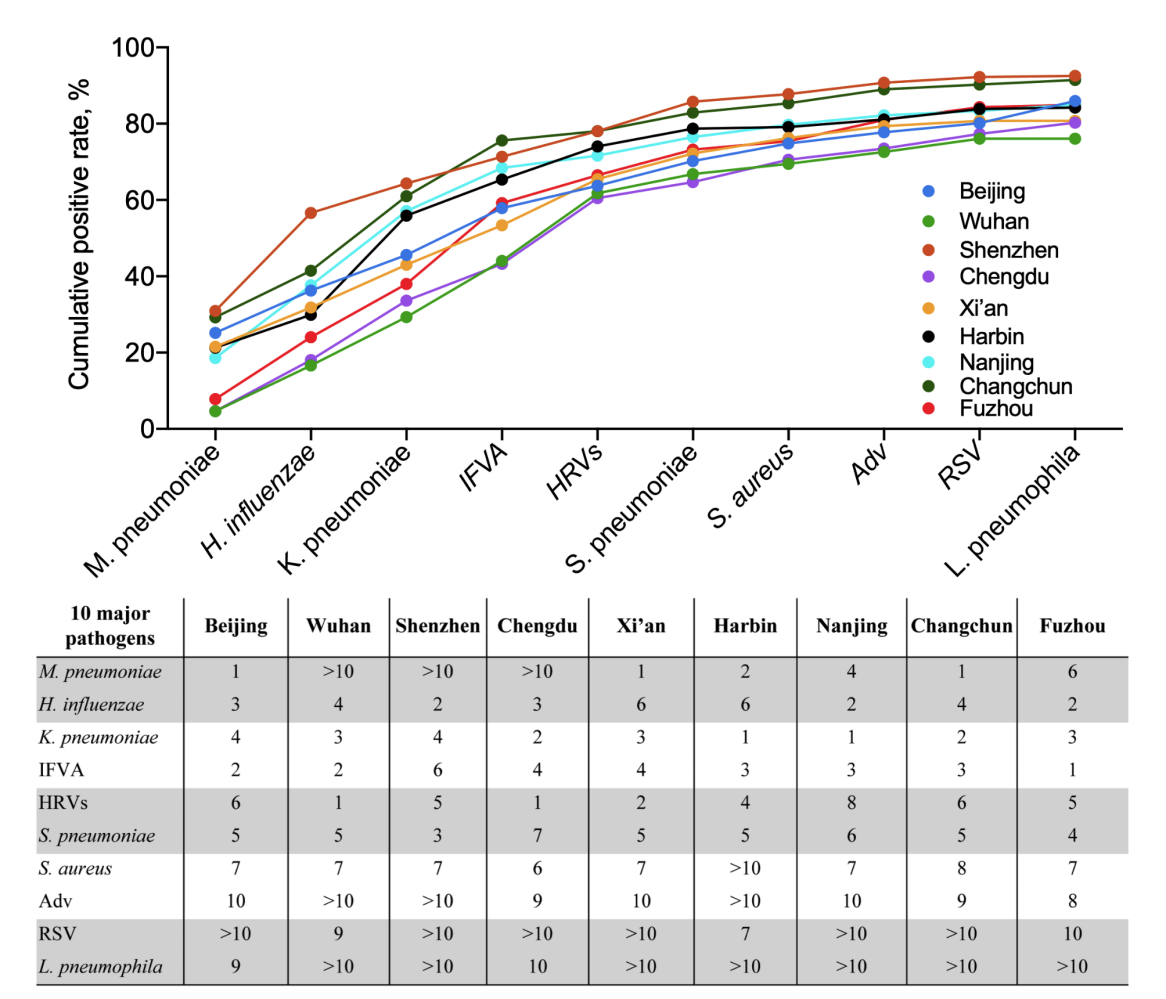
**Cumulative proportion of the ten major pathogens at each sentinel site in community-acquired pneumonia patients.** The line chart shows the cumulative positive rates of the ten major site-specific pathogens calculated by taking into account the detection rate and the risk of severe illness according to the site-combined data. The number in the box indicates the ranking of the pathogen detected at that site

Considering the overall positive detection rate and the associations with severe infections, we proposed that the ten major pathogens according to the aetiological estimation fraction, including the top eight most frequently detected pathogens, RSV and *L. pneumophila*, accounting for 76.06–92.52% of all positive detection results across sites, should be given priority in screening (Fig. 5, Additional file 11: Table S8).

## Discussion

In this study, we identified the respiratory pathogens spectra in adults with CAP in China via a multicentre prospective study. Our data showed that the pathogen spectra were consistent across different geographical regions over years. We further demonstrated that 10 major pathogens account for 76.06–92.52% of all positive detections across sites. *K. pneumoniae*, *S. aureus*, IFVA and RSV were risk factors related to severe CAP. These findings update our understanding of the aetiology of CAP in China, which may largely inform the development clinical pathogen diagnosis, anti-infective intervention, and even vaccination.

Molecular testing method has been shown to improve pathogen diagnosis efficiency and feasibility according to several large-scale studies on the aetiology of CAP [5, 15–18]. By using the RT‒PCR method, the overall positive detection rate of common respiratory pathogens in our study was 60.36%, which is comparable with previous reports, for example, in Africa (59.6%), Europe and the United States (60.6%) [16, 17], demonstrating the reliability of molecular test methods. IFVs, *S. pneumoniae*, *H. influenzae*, *M. pneumoniae* and HRVs were considered the major detected CAP pathogens [8, 9, 17–20]. According to our results, the viral spectrum was similar to that in previous studies, but the dominant nonviral agents varied compared with the data from other countries. For example, *S. pneumoniae* and *M. pneumoniae* were found at high frequencies in adults with CAP in USA, Finland and Australia, and *S. pneumoniae* and *H. influenzae* were the most frequently detected bacteria in adults with CAP in Sweden, Japan and Chile [5, 19–23], while *M. pneumoniae* and *K. pneumoniae* were found at high frequencies in our study. In addition, *K. pneumoniae* was also identified as the fourth most commonly detected pathogen in patients with acute respiratory infection in China [24], reflecting the regional distribution of the pathogens. The frequency of detected pathogens may be affected by multiple factors, including medical, social, economic, environmental, geographical, and demographic factors. The difference in the implementation of national immunization programs and the use of antibiotics may also influence the aetiology of respiratory infections.

Elucidating the roles of different pathogens in severe CAP is critical for identifying the risk factors for severe infections to improve disease management. However, confounding factors might influence this determination [25]. In this study, after adjusting for confounding factors, including age, sex, season, days post symptom onset, underlying diseases, and antibiotic exposure before admission, IFVA, *K. pneumoniae*, *S. aureus*, and RSV were found to be strongly associated with severe infections, suggesting the important aetiological roles of these pathogens in severe CAP. *K. pneumonia* was found to be correlated with adverse outcomes and had a high detection rate in our study. These results indicated that *K. pneumoniae* should be seriously considered as a priority screening pathogen in adults with CAP. Further investigations should be performed to determine the pathogenicity and virulence characteristics of *K. pneumoniae.*

Aetiological studies on respiratory infections have defined a broad range of pathogens. However, the prevalence of these dozens of pathogens including viruses, bacteria, fungi and parasites varies largely, indicating the differential role of the pathogens play in respiratory infections. It is hard and unnecessary to cover all the respiratory pathogens in clinic panel for pathogen diagnosis for benefit/cost and technical reasons. Therefore, precise definition is required to design a panel which can cover the majority of respiratory pathogens with appropriate cost based on studies on respiratory pathogen prevalence. Our data showed that the ten major pathogens in CAP, including *M. pneumoniae* (376, 11.05%), *H. influenzae* (363, 10.67%), *K. pneumoniae* (355, 10.43%), IFVA (323, 9.49%), HRVs (307, 9.02%), *S. pneumoniae* (253, 7.43%), *S. aureus* (153, 4.50%), Adv (100, 2.94%), RSV (80, 2.35%), and *L. pneumophila* (35, 1.03%), account for 76.06–92.52% of all positive detection results across sampling sites. These findings can inform the design of priority pathogen screening in China, which may increase the efficacy of common pathogen screening and decrease the unnecessary expenditure for aetiological diagnosis. Our data showed that *S. pneumoniae* and *H. influenzae* are among the top 10 aetiological agents detected in adults with CAP in our study. As the two vaccines have not been included in the national immunization program in China, our data strongly indicate the necessity to prioritize the inclusion of pneumococcal conjugate vaccines (PCVs) and Hib vaccines to mitigate the burden of the corresponding infections [26, 27].

Normally, the detection of *P. jirovecii* is mainly reported in immunocompromised hosts. However, serological tests support the possibility of subclinical infection, exposure, or fixation [28]. We detected *P. jirovecii* by using a molecular method, which might increase the positive detection rate. Nearly 60% (32 of 54) of patients were positive for multiple pathogens. We considered the positive detection of *P. jirovecii* to be reliable, while the clinical significance of the positive detection of *P. jirovecii* needs intensive investigation.

Our study had several limitations. Firstly, although the presence of pathogens determined by molecular testing method has been accepted, more comparative studies involving bacterial culture and other traditional methods used in the clinic are still needed to improve the strategies used for pathogen detection [29]. Especially, some respiratory samples were found to be positive on *L. pneumophila* in our study, but the clinical significance of the results still needs to be investigated intensively. However, very limited cases were tested by using urinary antigen detection for *L. pneumophila* in clinic. Secondly, the process for sampling sputum or BALF might introduce commensal bacterial contamination from the upper respiratory tract, which might influence the detection results. In addition, self-administration of antibiotics before admission was an unavoidable confounding factor in aetiology studies. Finally, our study was designed and performed before the COVID-19 pandemic, and our findings should be further compared with the aetiology of CAP post COVID-19.

## Conclusions

In conclusion, we clarified the pathogen spectrum in adults with CAP in China and characterized the pathogens associated with severe infection. On this basis, we propose to include 10 major pathogens as priorities for clinical pathogen screening in adults with CAP.

## Electronic supplementary material

Below is the link to the electronic supplementary material.

Supplementary Material 1

Supplementary Material 2

Supplementary Material 3

Supplementary Material 4

Supplementary Material 5

Supplementary Material 6

Supplementary Material 7

Supplementary Material 8

Supplementary Material 9

Supplementary Material 10

Supplementary Material 11

## Data Availability

The datasets used and/or analyzed during the current study are available from the corresponding author on reasonable request.
