# Supplementary material for: Identification of priority pathogens for aetiological diagnosis in adults with community-acquired pneumonia in China: a multicentre prospective study
Source: BMC Infect Dis. 2023 Apr 14;23:231. doi: 10.1186/s12879-023-08166-3 (PMC10103676; doi:10.1186/s12879-023-08166-3)
Supplement: Supplementary file 1 — Supplementary Material 1 [file 12879_2023_8166_MOESM1_ESM.docx]

**Additional file 1: Supplementary Methods**

**Enrolled criteria and exclusion criteria**

Community-acquired pneumonia **(**CAP) and severe CAP were diagnosed according to the guideline by the American Thoracic Society in 2007 [1]. In brief, all the patients were recruited if met the criteria 1 and 2, and any one of the criteria 3, including (1) Aged more than 14 years suffered acute fever with disease onset days less than 7 days on admission; (2) Chest radiographs revealed new patchy infiltrates, lobar/segmental consolidation, ground glass or interstitial changes, with or without pleural effusion; (3) Clinical manifestations related with pneumonia, including 1) cough, expectoration or aggravation of existing respiratory disease symptoms, with or without thick sputum/chest pain/dyspnea/hemoptysis; 2) signs of lung consolidation and/or wet rales; 3) peripheral blood leukocytes >10×10^9^/L or <4×10^9^/L, with or without left shift of nucleus.

The exclusion criteria included the following: (1) inability to obtain informed consent; (2) immunosuppression or tolerance, immune function-related gene defects, including but not limited to malignant tumour, organ transplantation, human immunodeficiency virus infection, and use of immunosuppressants for 30 consecutive days before onset; and (3) alternative diagnoses, including pulmonary tuberculosis, pulmonary tumour, noninfectious pulmonary interstitial disease, pulmonary oedema, atelectasis, pulmonary embolism, pulmonary eosinophilic infiltration and pulmonary vasculitis.

Severe CAP need to meet at least one of the major criteria, including invasive mechanical ventilation or septic shock with the need for vasopressors, or three or more of the minor criteria, including (1) respiratory rate ≥30 breaths/min, (2) arterial oxygen pressure/fraction of inspired oxygen (PaO2/FiO2) ratio ≤250, (3) multilobar infiltrates, (4) confusion/disorientation, (5) uremia (blood urea nitrogen level ≥20 mg/dL), (6) leukopeniac (white blood cell count <4000 cells/mm^3^), (7) thrombocytopenia (<100,000 cells/mm^3^), (8) hypothermia (core temperature <36℃), (9) hypotension requiring aggressive fluid resuscitation.

**Reference**s

[1] Mandell L, Wunderink R, An Z, Bartlett J, Campbell G, Dean N, et al. Infectious Diseases Society of America/American Thoracic Society consensus guidelines on the management of community acquired pneumonia in adults. Clin Infect Dis. 2007; 44 Suppl 2 : S2772.
