# Supplementary material for: Identification of priority pathogens for aetiological diagnosis in adults with community-acquired pneumonia in China: a multicentre prospective study
Source: BMC Infect Dis. 2023 Apr 14;23:231. doi: 10.1186/s12879-023-08166-3 (PMC10103676; doi:10.1186/s12879-023-08166-3)
Supplement: Supplementary file 2 — Supplementary Material 2 [file 12879_2023_8166_MOESM2_ESM.docx]

**Additional file 2: Table S1. Number of enrolled patients from 10 hospitals in nine sites in China from 2014 to 2019.**

| **Region** | **Site** | **Hospital** | **Enrolled cases** |
| --- | --- | --- | --- |
| Northeast | Harbin | The 2^nd^ Affiliated Hospital of Harbin Medical University | 472 |
|  | Changchun | The First Hospital of Jilin University | 104 |
| Northwest | Xi’an | The First Affiliated Hospital of Xi’an Jiaotong University | 561 |
|  |  | Xijing Hospital, Fourth Military Medical University | 38 |
| North | Beijing | Peking University People’s Hospital | 271 |
| Central | Wuhan | Tongji Hospital, Tongji Medical College of Huazhong University of Science & Technology | 441 |
| East | Nanjing | Zhongda Hospital, School of Medicine, Southeast University | 319 |
| South | Shenzhen | Shenzhen Third People's Hospital | 507 |
|  | Fuzhou | Fujian Provincial Hospital | 260 |
| Southwest | Chengdu | West China Hospital, Sichuan University | 430 |
