# Supplementary material for: Identification of priority pathogens for aetiological diagnosis in adults with community-acquired pneumonia in China: a multicentre prospective study
Source: BMC Infect Dis. 2023 Apr 14;23:231. doi: 10.1186/s12879-023-08166-3 (PMC10103676; doi:10.1186/s12879-023-08166-3)
Supplement: Supplementary file 3 — Supplementary Material 3 [file 12879_2023_8166_MOESM3_ESM.docx]

**Additional file 3: Table S2. Previous antibiotic exposure in community-acquired pneumonia (CAP).**

| **Antibiotics used** | | **Total** | **Nonsevere** | **Severe** | **P-value** |
| --- | --- | --- | --- | --- | --- |
| Number of detected |  | 3132^a^ | 2736 | 396 | - |
| Any Drugs |  | 720 (22.99) | 573 (20.94) | 147 (37.12) | **<0.001** |
| Quinolones (n=232) | Moxifloxacin | 85 (2.71) | 61 (2.23) | 24 (6.06) | **<0.001** |
|  | Levofloxacin | 78 (2.49) | 63 (2.30) | 15 (3.79) | 0.076 |
|  | Moxifloxacin hydrochloride | 59 (1.88) | 43 (1.57) | 16 (4.04) | **0.001** |
|  | Levofloxacin hydrochloride injection | 56 (1.79) | 13 (0.48) | 3 (0.76) | 0.444 |
| β-lactams (n=351) | Cephalosporins | 43 (1.37) | 49 (1.79) | 7 (1.77) | 0.974 |
|  | Sulperazon | 39 (1.25) | 22 (0.80) | 21 (5.30) | **<0.001** |
|  | Meropenem | 27 (0.86) | 10 (0.37) | 17 (4.29) | **<0.001** |
|  | Amoxicillin | 25 (0.80) | 25 (0.91) | 0 (0) | 0.064 |
|  | Imipenem | 22 (0.70) | 13 (0.48) | 9 (2.27) | **0.001** |
|  | Ceftriaxone sodium | 21 (0.67) | 20 (0.73) | 1 (0.25) | 0.505 |
|  | Cefuroxime | 21 (0.67) | 18 (0.66) | 3 (0.76) | 0.742 |
|  | Imipenem and cilastatin sodium for injection | 21 (0.67) | 7 (0.26) | 14 (3.54) | **<0.001** |
|  | Mezlocillin sodium and sulbactam sodium for injection | 21 (0.67) | 13 (0.48) | 4 (1.01) | 0.258 |
|  | Piperacillin sodium and tazobactam sodium for injection | 17 (0.54) | 5 (0.18) | 9 (2.27) | **<0.001** |
|  | Latamoxef | 14 (0.45) | 12 (0.44) | 1 (0.25) | 1 |
|  | Penicillin | 13 (0.42) | 12 (0.44) | 0 (0) | 0.383 |
|  | Cefdinir | 12 (0.38) | 15 (0.55) | 1 (0.25) | 0.71 |
|  | Cefixime | 16 (0.51) | 18 (0.66) | 1 (0.25) | 0.499 |
|  | Sulbactam and cefopcrazone | 19 (0.61) | 12 (0.44) | 4 (1.01) | 0.134 |
|  | Cefotaxime sodium | 16 (0.51) | 10 (0.37) | 5 (1.26) | **0.032** |
|  | Ceftazidime | 16 (0.51) | 9 (0.33) | 4 (1.01) | 0.071 |
|  | Ceftizoxime | 15 (0.48) | 8 (0.29) | 2 (0.51) | 0.367 |
| Macrolides (n=39) | Azithromycin | 13 (0.42) | 35 (1.28) | 4 (1.01) | 0.811 |
| Other drugs (n=207) | Oseltamivir, etc | 207 (6.61) | 167 (6.10) | 40 (10.10) | **0.003** |

^a^ Information of previous antibiotic exposure was missing for 271 cases, therefore the total number for study of antibiotics use was 3132.
