# Supplementary material for: Identification of priority pathogens for aetiological diagnosis in adults with community-acquired pneumonia in China: a multicentre prospective study
Source: BMC Infect Dis. 2023 Apr 14;23:231. doi: 10.1186/s12879-023-08166-3 (PMC10103676; doi:10.1186/s12879-023-08166-3)
Supplement: Supplementary file 4 — Supplementary Material 4 [file 12879_2023_8166_MOESM4_ESM.docx]

**Additional file 4: Table S3. Detection rate of bacteria and viruses in community-acquired pneumonia (CAP).**

| **Pathogens** | **Positive detection** | **Single detection**^a^ | **Co-detection**^b^ | **Co-detection** | | | | |
| --- | --- | --- | --- | --- | --- | --- | --- | --- |
|  |  |  |  | **Dual** | **Triple** | | **Four and more** | |
| Total | 2054 (60.36)^c^ | 1329 (39.05) | 725 (21.30) | 542 (15.93) | | 145 (4.26) | | 38 (1.12) |
| Bacteria | 1400 (41.14) | 753 (22.13) | 647 (19.01) | 481 (14.13) | | 128 (3.76) | | 38 (1.12) |
| *M. pneumoniae* | 376 (11.05) | 243 (7.14) | 133 (3.91) | 101 (2.97) | | 26 (0.76) | | 6 (0.18) |
| *H. influenzae* | 363 (10.67) | 116 (3.41) | 239 (7.02) | 158 (4.64) | | 55 (1.62) | | 26 (0.76) |
| *K. pneumoniae* | 355 (10.43) | 151 (4.44) | 204 (5.99) | 136 (4.00) | | 49 (1.44) | | 19 (0.56) |
| *S. pneumoniae* | 253 (7.43) | 75 (2.20) | 178 (5.23) | 107 (3.14) | | 51 (1.50) | | 20 (0.59) |
| *S. aureus* | 153 (4.50) | 46 (1.35) | 107 (3.14) | 67 (1.97) | | 28 (0.82) | | 12 (0.35) |
| *M. catarrhalis* | 87 (2.56) | 31 (0.91) | 56 (1.65) | 30 (0.88) | | 22 (0.65) | | 4 (0.12) |
| *P. jirovecii* | 54 (1.59) | 22 (0.65) | 32 (0.94) | 23 (0.68) | | 6 (0.18) | | 3 (0.09) |
| *L. pneumophila* | 35 (1.03) | 24 (0.71) | 11 (0.32) | 8 (0.24) | | 3 (0.09) | | 0 (0) |
| *C. pneumoniae* | 35 (1.03) | 27 (0.79) | 8 (0.24) | 8 (0.24) | | 0 (0) | | 0 (0) |
| *Bordetella* spp | 27 (0.79) | 15 (0.44) | 12 (0.35) | 3 (0.09) | | 6 (0.18) | | 3 (0.09) |
| *Salmonella* spp | 3 (0.09) | 3 (0.09) | 0 (0) | 0 (0) | | 0 (0) | | 0 (0) |
| Viruses | 1106 (32.50) | 576 (16.93) | 530 (15.57) | 379 (11.14) | | 117 (3.44) | | 34 (1.00) |
| IFVA | 323 (9.49) | 158 (4.64) | 165 (4.85) | 111 (3.26) | | 32 (0.94) | | 22 (0.65) |
| HRVs | 307 (9.02) | 137 (4.03) | 170 (5.00) | 113 (3.32) | | 40 (1.18) | | 17 (0.50) |
| Adv | 100 (2.94) | 47 (1.38) | 53 (1.56) | 33 (0.97) | | 17 (0.50) | | 3 (0.09) |
| HPIV3 | 94 (2.76) | 32 (0.94) | 62 (1.82) | 27 (0.79) | | 23 (0.68) | | 12 (0.35) |
| RSV | 80 (2.35) | 42 (1.23) | 38 (1.12) | 25 (0.73) | | 11 (0.32) | | 2 (0.06) |
| IFVB | 74 (2.17) | 33 (0.97) | 41 (1.20) | 34 (1.00) | | 6 (0.18) | | 1 (0.03) |
| HMPV | 66 (1.94) | 33 (0.97) | 33 (0.97) | 24 (0.71) | | 8 (0.24) | | 1 (0.03) |
| HCoV-229E | 57 (1.67) | 24 (0.71) | 33 (0.97) | 15 (0.44) | | 14 (0.41) | | 4 (0.12) |
| HCoV-OC43 | 53 (1.56) | 25 (0.73) | 28 (0.82) | 19 (0.56) | | 7 (0.21) | | 2 (0.06) |
| HCoV-HKU1 | 28 (0.82) | 12 (0.35) | 16 (0.47) | 15 (0.44) | | 0 (0) | | 1 (0.03) |
| EVs | 26 (0.76) | 10 (0.29) | 16 (0.47) | 7 (0.21) | | 7 (0.21) | | 2 (0.06) |
| HCoV-NL63 | 24 (0.71) | 9 (0.26) | 15 (0.44) | 8 (0.24) | | 6 (0.18) | | 1 (0.03) |
| HPIV4 | 12 (0.35) | 4 (0.12) | 8 (0.24) | 3 (0.09) | | 3 (0.09) | | 2 (0.06) |
| HPIV1 | 10 (0.29) | 3 (0.09) | 7 (0.21) | 3 (0.09) | | 4 (0.12) | | 0 (0) |
| HPIV2 | 8 (0.24) | 4 (0.12) | 4 (0.12) | 2 (0.06) | | 2 (0.06) | | 0 (0) |
| HPeV | 5 (0.15) | 24 (0.71) | 5 (0.15) | 0 (0) | | 4 (0.12) | | 1 (0.03) |
| HBoV | 5 (0.15) | 2 (0.06) | 3 (0.09) | 1 (0.03) | | 2 (0.06) | | 0 (0) |
| IFVC | 1 (0.03) | 1 (0.03) | 0 (0) | 0 (0) | | 0 (0) | | 0 (0) |

^a^ Single detection means detection of a pathogen (a virus or a bacterium).

^b^ Co-detection means detection of multiple pathogens (two or more viruses or two or more bacteria or both viruses and bacteria).

^c^ The numbers in parentheses indicate the percentages of positive infection in total samples.

*H. Influenzae*, *Haemophilus influenzae*; *K. pneumoniae*, *Klebsiella pneumoniae*; *S. pneumoniae*, *Streptococcus pneumoniae*; *M. pneumoniae*, *Mycoplasma pneumoniae*; *S. aureus*, *Staphylococcus aureus*; *M. catarrhalis*, *Moraxella catarrhalis*; *P. jirovecii*, *Pneumocystis jirovecii*; *Bordetella* spp, *Bordetella* spp (expect *Bordetella parapertussis*; *L. pneumophila*, *Legionella pneumophila*/*Legionella longbeachae*; *C. pneumonia*, *Chlamydia pneumoniae*; *Hib*, *Haemophilus influenzae type B*; *Salmonella* spp, *Salmonella* species; HRV, human rhinoviruses; IFVs, influenza viruses; HPIVs, parainfluenza viruses; Adv, adenovirus; RSV, respiratory syncytial virus; HMPV, human metapneumovirus; HCoVs, human coronaviruses; HBoV, human bocavirus; EVs, enteroviruses.
