# Supplementary material for: Identification of priority pathogens for aetiological diagnosis in adults with community-acquired pneumonia in China: a multicentre prospective study
Source: BMC Infect Dis. 2023 Apr 14;23:231. doi: 10.1186/s12879-023-08166-3 (PMC10103676; doi:10.1186/s12879-023-08166-3)
Supplement: Supplementary file 5 — Supplementary Material 5 [file 12879_2023_8166_MOESM5_ESM.docx]

**Additional file 5: Figure S1. The pathogen detection rates without viral subtype among Chinese adults with community-acquired pneumonia (CAP)**. The dark shade represents single detection, while the light shade represents co-detection.

**、
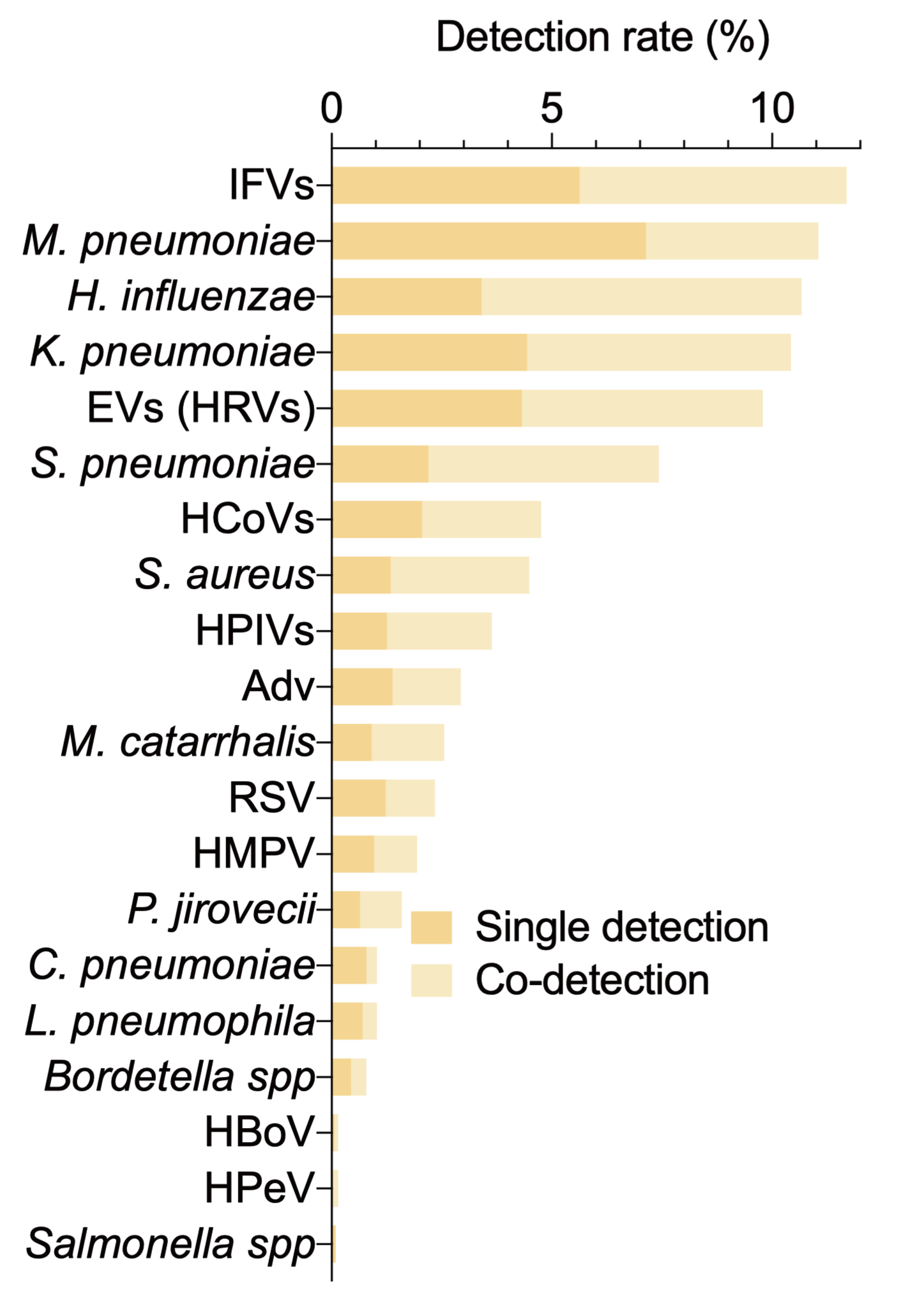
**
