# Supplementary material for: Identification of priority pathogens for aetiological diagnosis in adults with community-acquired pneumonia in China: a multicentre prospective study
Source: BMC Infect Dis. 2023 Apr 14;23:231. doi: 10.1186/s12879-023-08166-3 (PMC10103676; doi:10.1186/s12879-023-08166-3)
Supplement: Supplementary file 6 — Supplementary Material 6 [file 12879_2023_8166_MOESM6_ESM.docx]

**Additional file 6: Table S4. Detection of respiratory pathogens in Chinese adults with community-acquired pneumonia (CAP) by site.**

| **Site, no (%)** | **Beijing (n=271)** | **Wuhan (n=441)** | **Shenzhen (n=507)** | **Chengdu (n=430)** | **Xi’an (n=599)** | **Harbin (n=472)** | **Nanjing(n=319)** | **Changchun (n=104)** | **Fuzhou (n=260)** |
| --- | --- | --- | --- | --- | --- | --- | --- | --- | --- |
| Positive detection | 171 (63.10)^a^ | 259 (58.73) | 401 (79.09) | 238 (55.35) | 223 (37.23) | 254 (53.81) | 247 (77.43) | 82 (78.85) | 179 (68.85) |
| Bacteria | 125 (46.13) | 129 (29.25) | 332 (65.48) | 146 (33.95) | 141 (23.54) | 173 (36.65) | 192 (60.19) | 61 (58.65) | 101 (38.85) |
| *M. pneumoniae* | 43 (15.87) | 12 (2.72) | 124 (24.46) | 11 (2.56) | 48 (8.01) | 54 (11.44) | 46 (14.42) | 24 (23.08) | 14 (5.38) |
| *H. influenzae* | 19 (7.01) | 31 (7.03) | 121 (23.87) | 33 (7.67) | 25 (4.17) | 24 (5.08) | 55 (17.24) | 12 (11.54) | 31 (11.92) |
| *K. pneumoniae* | 17 (6.27) | 37 (8.39) | 46 (9.07) | 40 (9.30) | 28 (4.67) | 72 (15.25) | 65 (20.38) | 22 (21.15) | 28 (10.77) |
| *S. pneumoniae* | 17 (6.27) | 26 (5.90) | 68 (13.41) | 21 (4.88) | 26 (4.34) | 27 (5.72) | 31 (9.72) | 12 (11.54) | 25 (9.62) |
| *S. aureus* | 11 (4.06) | 17 (3.85) | 39 (7.69) | 23 (5.35) | 14 (2.34) | 7 (1.48) | 24 (7.52) | 5 (4.81) | 13 (5.00) |
| *M. catarrhalis* | 0 (0) | 16 (3.63) | 21 (4.14) | 8 (1.86) | 10 (1.67) | 8 (1.69) | 15 (4.70) | 3 (2.88) | 6 (2.31) |
| *P. jirovecii* | 0 (0) | 15 (3.40) | 2 (0.39) | 24 (5.58) | 4 (0.67) | 4 (0.85) | 3 (0.94) | 0 (0) | 2 (0.77) |
| *L. pneumophila* | 10 (3.69) | 1 (0.23) | 3 (0.59) | 13 (3.02) | 0 (0) | 2 (0.42) | 4 (1.25) | 1 (0.96) | 1 (0.38) |
| *C. pneumoniae* | 10 (3.69) | 0 (0) | 10 (1.97) | 0 (0) | 6 (1.00) | 3 (0.64) | 5 (1.57) | 0 (0) | 1 (0.38) |
| *Bordetella* spp | 0 (0) | 4 (0.91) | 7 (1.38) | 2 (0.47) | 2 (0.33) | 3 (0.64) | 4 (1.25) | 3 (2.88) | 2 (0.77) |
| *Hib* | 0 (0) | 3 (0.68) | 0 (0) | 0 (0) | 4 (0.67) | 2 (0.42) | 3 (0.94) | 0 (0) | 0 (0) |
| *Salmonella* spp | 0 (0) | 0 (0) | 0 (0) | 2 (0.47) | 0 (0) | 1 (0.21) | 0 (0) | 0 (0) | 0 (0) |
| Viruses | 72 (26.57) | 186 (42.18) | 159 (31.36) | 143 (33.26) | 118 (19.70) | 136 (28.81) | 129 (40.44) | 47 (45.19) | 116 (44.62) |
| HRVs | 16 (5.90) | 66 (14.97) | 46 (9.07) | 51 (11.86) | 38 (6.34) | 38 (8.05) | 23 (7.21) | 10 (9.62) | 19 (7.31) |
| IFVA | 28 (10.33) | 43 (9.75) | 46 (9.07) | 27 (6.28) | 28 (4.67) | 39 (8.26) | 48 (15.05) | 17 (16.35) | 47 (18.08) |
| IFVB | 6 (2.21) | 15 (3.40) | 16 (3.16) | 8 (1.86) | 5 (0.83) | 5 (1.06) | 1 (0.31) | 7 (6.73) | 11 (4.23) |
| IFVC | 0 (0) | 0 (0) | 0 (0) | 1 (0.23) | 0 (0) | 0 (0) | 0 (0) | 0 (0) | 0 (0) |
| Adv | 7 (2.58) | 15 (3.40) | 19 (3.75) | 15 (3.49) | 9 (1.50) | 7 (1.48) | 12 (3.76) | 4 (3.85) | 12 (4.62) |
| RSV | 4 (1.48) | 16 (3.63) | 12 (2.37) | 10 (2.33) | 7 (1.17) | 14 (2.97) | 6 (1.88) | 2 (1.92) | 9 (3.46) |
| HCoV-229E | 0 (0) | 21 (4.76) | 1 (0.20) | 18 (4.19) | 4 (0.67) | 8 (1.69) | 3 (0.94) | 1 (0.96) | 1 (0.38) |
| HCoV-OC43 | 2 (0.74) | 8 (1.81) | 6 (1.18) | 4 (0.93) | 8 (1.34) | 9 (1.91) | 7 (2.19) | 2 (1.92) | 7 (2.69) |
| HCoV-HKU1 | 1 (0.37) | 3 (0.68) | 2 (0.39) | 4 (0.93) | 3 (0.50) | 10 (2.12) | 2 (0.63) | 2 (1.92) | 1 (0.38) |
| HCoV-NL63 | 0 (0) | 2 (0.45) | 2 (0.39) | 2 (0.47) | 12 (2.00) | 6 (1.27) | 0 (0) | 0 (0) | 0 (0) |
| HPIV1 | 1 (0.37) | 6 (1.36) | 0 (0) | 3 (0.70) | 0 (0) | 0 (0) | 0 (0) | 0 (0) | 0 (0) |
| HPIV2 | 0 (0) | 2 (0.45) | 1 (0.20) | 2 (0.47) | 0 (0) | 2 (0.42) | 0 (0) | 0 (0) | 1 (0.38) |
| HPIV3 | 3 (1.11) | 11 (2.49) | 4 (0.79) | 6 (1.40) | 9 (1.50) | 5 (1.06) | 46 (14.42) | 3 (2.88) | 7 (2.69) |
| HPIV4 | 1 (0.37) | 2 (0.45) | 4 (0.79) | 0 (0) | 1 (0.17) | 0 (0) | 2 (0.63) | 0 (0) | 2 (0.77) |
| HMPV | 2 (0.74) | 10 (2.27) | 7 (1.38) | 12 (2.79) | 9 (1.50) | 9 (1.91) | 10 (3.13) | 1 (0.96) | 6 (2.31) |
| EVs | 1 (0.37) | 2 (0.45) | 3 (0.59) | 6 (1.40) | 1 (0.17) | 3 (0.64) | 3 (0.94) | 3 (2.88) | 4 (1.54) |
| HBoV | 1 (0.37) | 0 (0) | 0 (0) | 1 (0.23) | 0 (0) | 0 (0) | 1 (0.31) | 1 (0.96) | 1 (0.38) |
| HPeV | 0 (0) | 0 (0) | 0 (0) | 1 (0.23) | 1 (0.17) | 1 (0.21) | 2 (0.63) | 0 (0) | 0 (0) |

^a^ Numbers in parentheses indicate the percentage of positive infection in the total samples for that site.
