# Supplementary material for: Identification of priority pathogens for aetiological diagnosis in adults with community-acquired pneumonia in China: a multicentre prospective study
Source: BMC Infect Dis. 2023 Apr 14;23:231. doi: 10.1186/s12879-023-08166-3 (PMC10103676; doi:10.1186/s12879-023-08166-3)
Supplement: Supplementary file 7 — Supplementary Material 7 [file 12879_2023_8166_MOESM7_ESM.docx]

**Additional file 7: Table S5. Age distribution of patients with community-acquired pneumonia (CAP).**

| **Age group, no (%)** | **14-24 yrs (n=231)** | **25-44 yrs (n=795)** | **45-64 yrs (n=1161)** | **≥65 yrs (n=1216)** | **P-value**^b^ |
| --- | --- | --- | --- | --- | --- |
| Positive cases | 160 (69.26)^a^ | 530 (66.67) | 638 (54.95) | 726 (59.70) | **0.001** |
| Bacteria | 130 (56.28) | 401 (50.44) | 410 (35.31) | 459 (37.75) | **0.001** |
| *M. pneumoniae* | 72 (31.17) | 183 (23.02) | 69 (5.94) | 52 (4.28) | **0.001** |
| *H. influenzae* | 23 (9.96) | 93 (11.70) | 114 (9.82) | 121 (9.95) | 0.540 |
| *K. pneumoniae* | 13 (5.63) | 71 (8.93) | 111 (9.56) | 160 (13.16) | **0.001** |
| *S. pneumoniae* | 21 (9.09) | 49 (6.16) | 92 (7.92) | 91 (7.48) | 0.362 |
| *S. aureus* | 14 (6.06) | 31 (3.90) | 54 (4.65) | 54 (4.44) | 0.562 |
| *M. catarrhalis* | 4 (1.73) | 17 (2.14) | 31 (2.67) | 35 (2.88) | 0.625 |
| *P. jirovecii* | 2 (0.87) | 9 (1.13) | 21 (1.81) | 22 (1.81) | 0.462 |
| *L. pneumophila* | 2 (0.87) | 3 (0.38) | 17 (1.46) | 13 (1.07) | 0.135 |
| *C. pneumoniae* | 6 (2.60) | 19 (2.39) | 7 (0.60) | 3 (0.25) | **0.001** |
| *Bordetella* spp | 1 (0.43) | 7 (0.88) | 11 (0.95) | 8 (0.66) | 0.779 |
| *Hib* | 0 (0) | 5 (0.63) | 6 (0.52) | 1 (0.08) | 0.113 |
| *Salmonella* spp | 1 (0.43) | 0 (0) | 0 (0) | 2 (0.16) | 0.130 |
| Viruses | 57 (24.68) | 248 (31.19) | 377 (32.47) | 424 (34.87) | **0.017** |
| HRVs | 24 (10.39) | 70 (8.81) | 104 (8.96) | 109 (8.96) | 0.900 |
| IFVA | 7 (3.03) | 73 (9.18) | 121 (10.42) | 122 (10.03) | **0.005** |
| IFVB | 3 (1.30) | 12 (1.51) | 23 (1.98) | 36 (2.96) | 0.101 |
| IFVC | 0 (0) | 0 (0) | 0 (0) | 1 (0.08) | 0.615 |
| Adv | 10 (4.33) | 31 (3.90) | 26 (2.24) | 33 (2.71) | 0.096 |
| RSV | 0 (0) | 14 (1.76) | 33 (2.84) | 33 (2.71) | **0.034** |
| HCoV-229E | 2 (0.87) | 10 (1.26) | 27 (2.33) | 18 (1.48) | 0.170 |
| HCoV-OC43 | 3 (1.30) | 11 (1.38) | 20 (1.72) | 19 (1.56) | 0.927 |
| HCoV-HKU1 | 4 (1.73) | 6 (0.75) | 7 (0.60) | 11 (0.90) | 0.366 |
| HCoV-NL63 | 2 (0.87) | 4 (0.50) | 9 (0.78) | 9 (0.74) | 0.885 |
| HPIV1 | 0 (0) | 1 (0.13) | 5 (0.43) | 4 (0.33) | 0.524 |
| HPIV2 | 2 (0.87) | 0 (0) | 2 (0.17) | 4 (0.33) | 0.092 |
| HPIV3 | 2 (0.87) | 16 (2.01) | 32 (2.76) | 44 (3.62) | **0.044** |
| HPIV4 | 1 (0.43) | 3 (0.38) | 4 (0.34) | 4 (0.33) | 0.994 |
| HMPV | 4 (1.73) | 15 (1.89) | 22 (1.89) | 25 (2.06) | 0.983 |
| EVs | 2 (0.87) | 5 (0.63) | 9 (0.78) | 10 (0.82) | 0.964 |
| HBoV | 0 (0) | 2 (0.25) | 0 (0) | 3 (0.25) | 0.325 |
| HPeV | 0 (0) | 2 (0.25) | 1 (0.09) | 2 (0.16) | 0.741 |

^a^ Numbers in parentheses indicate the percentage of positive infection in the total samples for that age group.

^b^ The chi-square test was used for comparison of the detection rate in different age groups and p<0.05 was considered statistically significant
