# Supplementary material for: Identification of priority pathogens for aetiological diagnosis in adults with community-acquired pneumonia in China: a multicentre prospective study
Source: BMC Infect Dis. 2023 Apr 14;23:231. doi: 10.1186/s12879-023-08166-3 (PMC10103676; doi:10.1186/s12879-023-08166-3)
Supplement: Supplementary file 8 — Supplementary Material 8 [file 12879_2023_8166_MOESM8_ESM.docx]

**Additional file 8: Table S6. Annual distribution of respiratory pathogens in community-acquired pneumonia (CAP).**

| **Pathogens, no (%)** | **2014 (n=327)** | **2015 (n=739)** | **2016 (n=582)** | **2017 (n=788)** | **2018 (n=522)** | **2019 (n=445)** |
| --- | --- | --- | --- | --- | --- | --- |
| Positive detection | 237 (72.48)^a^ | 444 (60.08) | 265 (45.53) | 493 (62.56) | 322 (61.69) | 281 (63.15) |
| Bacteria | 137 (41.90) | 204 (27.60) | 119 (20.45) | 199 (25.25) | 164 (31.42) | 119 (26.74) |
| *M. pneumoniae* | 55 (16.82) | 69 (9.34) | 46 (7.90) | 96 (12.18) | 50 (9.58) | 63 (14.16) |
| *H. influenzae* | 40 (12.23) | 74 (10.01) | 47 (8.08) | 80 (10.15) | 54 (10.34) | 52 (11.69) |
| *K. pneumoniae* | 64 (19.57) | 82 (11.10) | 26 (4.47) | 76 (9.64) | 51 (9.77) | 54 (12.13) |
| *S. pneumoniae* | 21 (6.42) | 53 (7.17) | 25 (4.30) | 73 (9.26) | 55 (10.54) | 25 (5.62) |
| *S. aureus* | 28 (8.56) | 37 (5.01) | 16 (2.75) | 26 (3.30) | 29 (5.56) | 15 (3.37) |
| *M. catarrhalis* | 12 (3.67) | 20 (2.71) | 20 (3.44) | 18 (2.28) | 8 (1.53) | 9 (2.02) |
| *P. jirovecii* | 5 (1.53) | 26 (3.52) | 10 (1.72) | 8 (1.02) | 5 (0.96) | 0 (0.00) |
| *L. pneumophila* | 4 (1.22) | 2 (0.27) | 12 (2.06) | 5 (0.63) | 10 (1.92) | 2 (0.45) |
| *C. pneumoniae* | 6 (1.83) | 2 (0.27) | 2 (0.34) | 11 (1.40) | 12 (2.30) | 2 (0.45) |
| *Bordetella* spp | 0 (0.00) | 8 (1.08) | 3 (0.52) | 4 (0.51) | 6 (1.15) | 6 (1.35) |
| *Hib* | 5 (1.53) | 1 (0.14) | 0 (0.00) | 5 (0.63) | 1 (0.19) | 0 (0.00) |
| *Salmonella* spp | 0 (0.00) | 3 (0.41) | 0 (0.00) | 0 (0.00) | 0 (0.00) | 0 (0.00) |
| Viruses | 62 (18.96) | 144 (19.49) | 96 (16.49) | 158 (20.05) | 93 (17.82) | 95 (21.35) |
| HRVs | 35 (10.70) | 65 (8.80) | 44 (7.56) | 74 (9.39) | 41 (7.85) | 44 (9.89) |
| IFVA | 26 (7.95) | 54 (7.31) | 29 (4.98) | 104 (13.20) | 50 (9.58) | 60 (13.48) |
| IFVB | 2 (0.61) | 18 (2.44) | 10 (1.72) | 18 (2.28) | 18 (3.45) | 8 (1.80) |
| IFVC | 0 (0.00) | 0 (0.00) | 1 (0.17) | 0 (0.00) | 0 (0.00) | 0 (0.00) |
| Adv | 12 (3.67) | 23 (3.11) | 12 (2.06) | 24 (3.05) | 12 (2.30) | 15 (3.37) |
| RSV | 12 (3.67) | 20 (2.71) | 9 (1.55) | 14 (1.78) | 12 (2.30) | 13 (2.92) |
| HCoV-229E | 3 (0.92) | 23 (3.11) | 9 (1.55) | 18 (2.28) | 1 (0.19) | 3 (0.67) |
| HCoV-OC43 | 4 (1.22) | 10 (1.35) | 11 (1.89) | 19 (2.41) | 5 (0.96) | 4 (0.90) |
| HCoV-HKU1 | 3 (0.92) | 8 (1.08) | 2 (0.34) | 7 (0.89) | 4 (0.77) | 4 (0.90) |
| HCoV-NL63 | 12 (3.67) | 5 (0.68) | 2 (0.34) | 1 (0.13) | 4 (0.77) | 0 (0.00) |
| HPIV1 | 1 (0.31) | 6 (0.81) | 1 (0.17) | 1 (0.13) | 0 (0.00) | 1 (0.22) |
| HPIV2 | 0 (0.00) | 2 (0.27) | 1 (0.17) | 5 (0.63) | 0 (0.00) | 0 (0.00) |
| HPIV3 | 4 (1.22) | 8 (1.08) | 10 (1.72) | 56 (7.11) | 8 (1.53) | 8 (1.80) |
| HPIV4 | 5 (1.53) | 1 (0.14) | 1 (0.17) | 2 (0.25) | 2 (0.38) | 1 (0.22) |
| HMPV | 4 (1.22) | 28 (3.79) | 9 (1.55) | 9 (1.14) | 10 (1.92) | 6 (1.35) |
| EVs | 4 (1.22) | 3 (0.41) | 7 (1.20) | 1 (0.13) | 3 (0.57) | 8 (1.80) |
| HBoV | 0 (0.00) | 1 (0.14) | 0 (0.00) | 1 (0.13) | 1 (0.19) | 2 (0.45) |
| HPeV | 2 (0.61) | 1 (0.14) | 2 (0.34) | 0 (0.00) | 0 (0.00) | 0 (0.00) |

^a^ Numbers in parentheses indicate the percentage of positive infection in the total samples for that year.
