# Supplementary material for: Identification of priority pathogens for aetiological diagnosis in adults with community-acquired pneumonia in China: a multicentre prospective study
Source: BMC Infect Dis. 2023 Apr 14;23:231. doi: 10.1186/s12879-023-08166-3 (PMC10103676; doi:10.1186/s12879-023-08166-3)
Supplement: Supplementary file 9 — Supplementary Material 9 [file 12879_2023_8166_MOESM9_ESM.docx]

**Additional file 9: Table S7. Seasonal distribution of respiratory pathogens in community-acquired pneumonia (CAP).**

| **Detection rates, no (%)** | **Spring (n=799)** | **Summer (n=753)** | **Autumn (n=911)** | **Winter (n=940)** | **P-value**^b^ |
| --- | --- | --- | --- | --- | --- |
| Positive detection | 473 (59.20)^a^ | 444 (58.96) | 557 (61.14) | 568 (60.43) | 0.773 |
| Bacteria | 214 (26.78) | 252 (33.47) | 287 (31.50) | 189 (20.11) | **<0.001** |
| *M. pneumoniae* | 52 (6.51) | 102 (13.55) | 148 (16.25) | 77 (8.19) | **<0.001** |
| 1. *influenzae* | 83 (10.39) | 84 (11.16) | 88 (9.66) | 92 (9.79) | 0.742 |
| *K. pneumoniae* | 69 (8.64) | 106 (14.08) | 112 (12.29) | 66 (7.02) | **<0.001** |
| 1. *pneumoniae* | 58 (7.26) | 61 (8.10) | 53 (5.82) | 80 (8.51) | 0.134 |
| *S. aureus* | 28 (3.50) | 36 (4.78) | 46 (5.05) | 41 (4.36) | 0.446 |
| *M. catarrhalis* | 19 (2.38) | 12 (1.59) | 35 (3.84) | 21 (2.23) | **0.025** |
| 1. *jirovecii* | 23 (2.88) | 12 (1.59) | 9 (0.99) | 10 (1.06) | **0.006** |
| *L. pneumophila* | 14 (1.75) | 10 (1.33) | 3 (0.33) | 8 (0.85) | 0.024 |
| *C. pneumoniae* | 9 (1.13) | 9 (1.20) | 6 (0.66) | 11 (1.17) | 0.639 |
| *Bordetella* spp | 7 (0.88) | 6 (0.80) | 10 (1.10) | 4 (0.43) | 0.431 |
| *Hib* | 2 (0.25) | 1 (0.13) | 6 (0.66) | 3 (0.32) | 0.292 |
| *Salmonella* spp | 1 (0.13) | 1 (0.13) | 0 (0) | 1 (0.11) | 0.769 |
| Viruses | 165 (20.65) | 102 (13.55) | 150 (16.47) | 231 (24.57) | **<0.001** |
| HRVs | 59 (7.38) | 43 (5.71) | 110 (12.07) | 91 (9.68) | **<0.001** |
| IFVA | 42 (5.26) | 76 (10.09) | 62 (6.81) | 143 (15.21) | **<0.001** |
| IFVB | 27 (3.38) | 5 (0.66) | 8 (0.88) | 34 (3.62) | **<0.001** |
| IFVC | 1 (0.13) | 0 (0) | 0 (0) | 0 (0) | 0.353 |
| Adv | 28 (3.50) | 14 (1.86) | 19 (2.09) | 37 (3.94) | **0.021** |
| RSV | 15 (1.88) | 9 (1.20) | 15 (1.65) | 41 (4.36) | **<0.001** |
| HCoV-229E | 14 (1.75) | 4 (0.53) | 16 (1.76) | 23 (2.45) | **0.024** |
| HCoV-OC43 | 19 (2.38) | 10 (1.33) | 19 (2.09) | 5 (0.53) | **0.008** |
| HCoV-HKU1 | 11 (1.38) | 5 (0.66) | 2 (0.22) | 10 (1.06) | **0.047** |
| HCoV-NL63 | 0 (0) | 6 (0.80) | 11 (1.21) | 7 (0.74) | **0.028** |
| HPIV1 | 5 (0.63) | 1 (0.13) | 1 (0.11) | 3 (0.32) | 0.191 |
| HPIV2 | 3 (0.38) | 0 (0) | 3 (0.33) | 2 (0.21) | 0.422 |
| HPIV3 | 28 (3.50) | 26 (3.45) | 22 (2.41) | 18 (1.91) | 0.117 |
| HPIV4 | 2 (0.25) | 3 (0.40) | 4 (0.44) | 3 (0.32) | 0.917 |
| HMPV | 27 (3.38) | 5 (0.66) | 11 (1.21) | 23 (2.45) | **<0.001** |
| EVs | 6 (0.75) | 5 (0.66) | 7 (0.77) | 8 (0.85) | 0.978 |
| HBoV | 2 (0.25) | 2 (0.27) | 1 (0.11) | 0 (0) | 0.428 |
| HPeV | 4 (0.50) | 0 (0) | 1 (0.11) | 0 (0) | 0.025 |

^a^ Numbers in parentheses indicate the percentage of positive infection in the total samples for that season.

^b^ The chi-square test was used for comparison of the detection rate in different seasons and p<0.05 was considered statistically significant.
