# Supplementary material for: Identification of priority pathogens for aetiological diagnosis in adults with community-acquired pneumonia in China: a multicentre prospective study
Source: BMC Infect Dis. 2023 Apr 14;23:231. doi: 10.1186/s12879-023-08166-3 (PMC10103676; doi:10.1186/s12879-023-08166-3)
Supplement: Supplementary file 10 — Supplementary Material 10 [file 12879_2023_8166_MOESM10_ESM.docx]

**Additional file 10: Figure S2. Co-detection patterns of respiratory pathogens in community-acquired pneumonia (CAP).** A. Co-detection with viruses and bacteria. B. Heatmap of the frequency for dual-detections. Each square represents the number of cases with two pathogens detected positive.

**
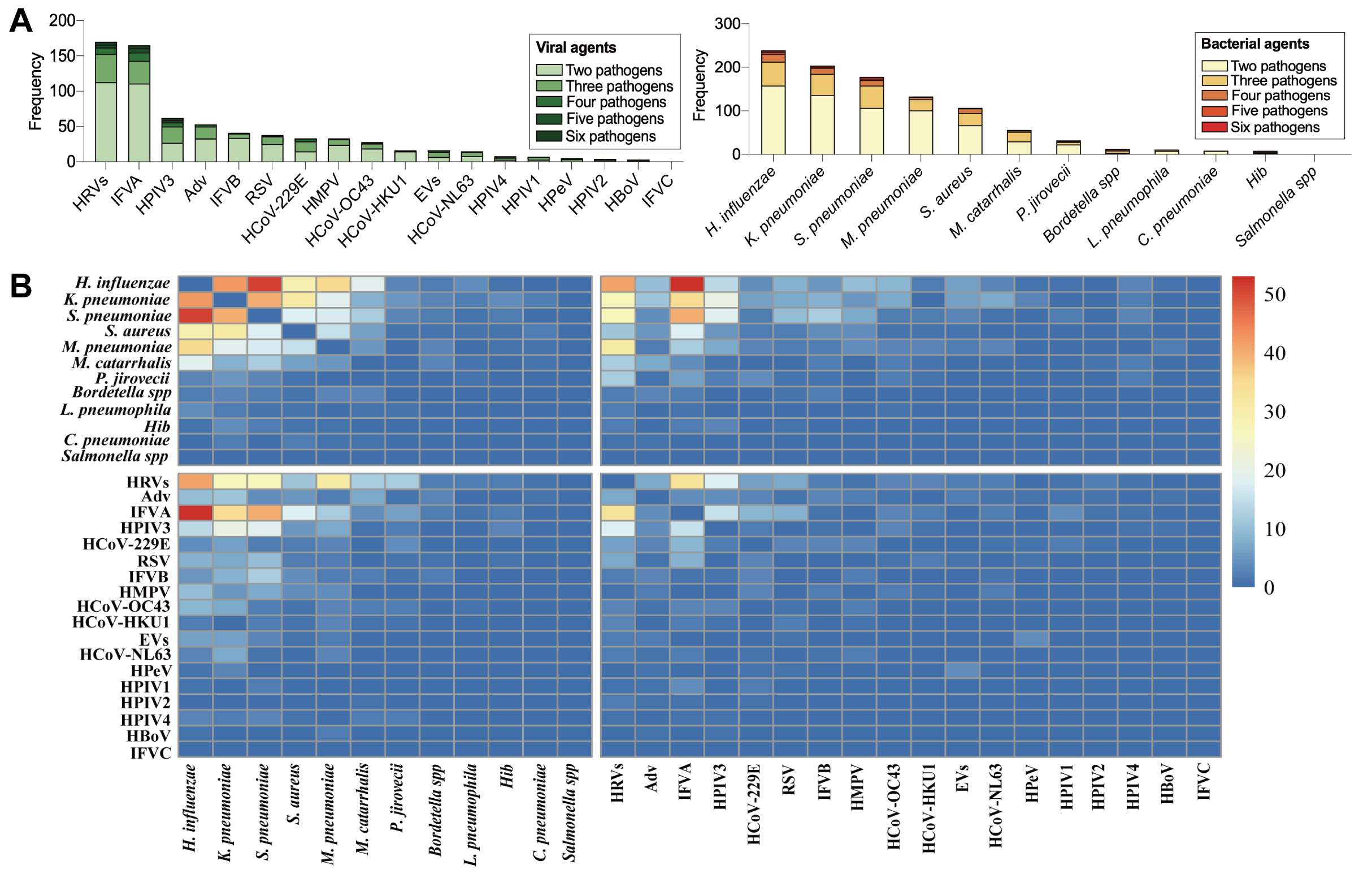
**
