# Supplementary material for: Identification of priority pathogens for aetiological diagnosis in adults with community-acquired pneumonia in China: a multicentre prospective study
Source: BMC Infect Dis. 2023 Apr 14;23:231. doi: 10.1186/s12879-023-08166-3 (PMC10103676; doi:10.1186/s12879-023-08166-3)
Supplement: Supplementary file 11 — Supplementary Material 11 [file 12879_2023_8166_MOESM11_ESM.docx]

**Additional file 11: Table S8. Aetiological estimated fraction for all pathogens in enrolled Chinese adults with community-acquired pneumonia (CAP).**

| **Order** | **Pathogens** | **Detection_rate (%)**^a^ | **Severe_OR**^b^ | **Aetiological estimated fraction (%)**^c^ |
| --- | --- | --- | --- | --- |
| 1 | IFVA | 9.49 | 2.77 | 26.29 |
| 2 | *K. pneumoniae* | 10.43 | 1.60 | 16.69 |
| 3 | HRVs | 9.02 | 1.00 | 9.02 |
| 4 | *S. aureus* | 4.50 | 1.88 | 8.46 |
| 5 | *S. pneumoniae* | 7.43 | 1.00 | 7.43 |
| 6 | RSV | 2.35 | 2.32 | 5.45 |
| 7 | *H. influenzae* | 10.67 | 0.47 | 5.01 |
| 8 | *L. pneumophila* | 1.03 | 4.09 | 4.21 |
| 9 | *M. pneumoniae* | 11.05 | 0.35 | 3.87 |
| 10 | Adv | 2.94 | 1.00 | 2.94 |
| 11 | HPIV3 | 2.76 | 1.00 | 2.76 |
| 12 | *M. catarrhalis* | 2.56 | 1.00 | 2.56 |
| 13 | IFVB | 2.17 | 1.00 | 2.17 |
| 14 | HMPV | 1.94 | 1.00 | 1.94 |
| 15 | HCoV-229E | 1.67 | 1.00 | 1.67 |
| 16 | *P. jirovecii* | 1.59 | 1.00 | 1.59 |
| 17 | HCoV-OC43 | 1.56 | 1.00 | 1.56 |
| 18 | *C. pneumoniae* | 1.03 | 1.00 | 1.03 |
| 19 | HCoV-HKU1 | 0.82 | 1.00 | 0.82 |
| 20 | *Bordetella* spp | 0.79 | 1.00 | 0.79 |
| 21 | EVs | 0.76 | 1.00 | 0.76 |
| 22 | HCoV-NL63 | 0.71 | 1.00 | 0.71 |
| 23 | HPIV4 | 0.35 | 1.00 | 0.35 |
| 24 | HPIV1 | 0.29 | 1.00 | 0.29 |
| 25 | HPIV2 | 0.24 | 1.00 | 0.24 |
| 26 | HPeV | 0.15 | 1.00 | 0.15 |
| 27 | HBoV | 0.15 | 1.00 | 0.15 |
| 28 | *Salmonella* spp | 0.09 | 1.00 | 0.09 |
| 29 | IFVC | 0.03 | 1.00 | 0.03 |

^a^ Detection rates were the result of combining data from all sites.

^b^ Odds ratios (ORs) adjusted by age (years), sex, season, duration of illness (days), previous antibiotic exposure and underlying diseases. Only those ORs with statistical significance were considered, and all those without significance were assigned a value of 1.00.

^c^ Aetiological estimated fraction (%) = Detection_rate (%) × Severe_OR.
